# Choroidal Changes After Anti-VEGF Therapy in AMD Eyes With Different Types of Macular Neovascularization Using Swept-Source OCT Angiography

**DOI:** 10.1167/iovs.64.13.16

**Published:** 2023-10-11

**Authors:** Mengxi Shen, Hao Zhou, Jie Lu, Jianqing Li, Xiaoshuang Jiang, Omer Trivizki, Rita Laiginhas, Jeremy Liu, Qinqin Zhang, Luis de Sisternes, William J. Feuer, Robert O'Brien, Giovanni Gregori, Ruikang K. Wang, Philip J. Rosenfeld

**Affiliations:** 1 Department of Ophthalmology, Bascom Palmer Eye Institute, University of Miami Miller School of Medicine, Miami, Florida, United States; 2 Department of Bioengineering, University of Washington, Seattle, Washington, United States; 3 Research and Development, Carl Zeiss Meditec, Inc., Dublin, California, United States

**Keywords:** swept-source optical coherence tomography angiography, age-related macular degeneration, optical coherence tomography, macular neovascularization, anti-VEGF

## Abstract

**Purpose:**

Choroidal changes before and after anti-VEGF therapy were investigated in eyes with exudative AMD to determine if there was a difference between eyes with macular neovascularization (MNV) that arises from the choroid (type 1 or 2) versus the retinal circulation (type 3).

**Methods:**

Patients with treatment-naïve AMD were imaged with swept-source optical coherence tomography angiography using a 12 × 12-mm scan pattern. The mean choroidal thickness and choroidal vascularity index (CVI) were measured within 5-mm and 11-mm fovea-centered circles before, at the onset of, and after anti-VEGF therapy.

**Results:**

Forty-one eyes of 37 patients were included; 24 eyes with type 1 MNV, 4 eyes with type 2 MNV, and 13 eyes with type 3 MNV. Within the 5-mm and 11-mm circles, the mean choroidal thickness and CVI measurements increased from pretreatment to the onset of exudation (*P* ≤ 0.03). The mean choroidal thickness and CVI measurements decreased from the onset of exudation to after treatment (*P* < 0.001). No significant changes in mean choroidal thickness or CVI were observed when comparing measurements before or after treatment (*P* ≥ 0.38). No significant differences in mean choroidal thickness or CVI measurements were observed between eyes with type 1 or 2 MNV and type 3 MNV.

**Conclusions:**

In treatment-naïve AMD eyes with MNV, the choroidal thickness and vascularity increased at the onset of exudation and then decreased after anti-VEGF therapy. This finding suggests that these choroidal changes develop in response to the proangiogenic milieu before treatment and in response to treatment, regardless of the site of origin for the MNV.

Exudative age-related macular degeneration (eAMD), which arises owing to macular neovascularization (MNV), is a late stage of AMD and a leading cause of irreversible vision loss among the elderly worldwide.^1^^–^^3^ Exudation from MNV is treated by intravitreal injections of VEGF inhibitors.^4^ Anti-VEGF therapy usually starts with fixed monthly dosing to resolve the exudation followed by optical coherence tomography (OCT)-guided retreatment.^5^ The goal of therapy is to maintain a fluid-free macula. Concerns have been reported that anti-VEGF therapy could promote thinning of the choroid and increase the risk of macular atrophy.^6^^–^^8^ Although the choroid does seem to change after anti-VEGF therapy,^9^^–^^11^ the concern that this change in thickness might result in macular atrophy over 2 years has not been supported by a prospective study.^12^ However, this result does not rule out the possibility that chronic anti-VEGF therapy might promote macular atrophy, because histopathological studies have demonstrated that choroidal vascular depletion is present in early AMD and is progressive in advanced AMD.^13^^,^^14^

In eAMD, previous studies have shown that both choroidal thickness and choroidal vascularity increase when exudation from MNV develops^15^^,^^16^ and decrease after treatment with anti-VEGF injections.^9^^–^^11^ However, there are no reports following the same eyes from the time before exudation develops, to the onset of exudation, and then after exudation resolves after anti-VEGF therapy. Moreover, it is unknown if the choroidal changes depend on the different forms of MNV. Because type 1 and type 2 MNV arise from the choroidal circulation and type 3 MNV arises from the retinal circulation,^1^ it is possible that anti-VEGF therapy might impact the choroid differently compared with MNV that resides entirely within the retina (type 3 MNV).

A previous report hypothesized that choroid thinning after anti-VEGF therapy is due to the pharmacological effect of anti-VEGF therapy causing vasoconstriction within the choroid.^17^ However, we offered an alternative explanation in our study of eyes with polypoidal choroidal vasculopathy (PCV) undergoing anti-VEGF therapy.^18^ We proposed that PCV, which arises from the choroidal circulation, could serve as an arteriovenous shunt between the choroidal arterial and venous circulations with increased flow compared with the non-neovascular state owing to the lower resistance of PCV lesion compared with the choriocapillaris. After anti-VEGF therapy, we found a decrease in choroidal thickness that could be explained by decreased flow within the PCV lesion. Like PCV, both type 1 and type 2 MNV arise from the choroidal circulation, whereas type 3 MNV is an intraretinal lesion with no connection to the choroid based on both histopathology and OCT angiography (OCTA) imaging.^19^^–^^21^ It is also known that eyes with type 3 MNV have thinner choroidal thickness measurements compared with both normal eyes and eyes with type 1 and type 2 MNV, which may impact pretreatment and post-treatment choroidal measurements.^22^^–^^24^ Therefore, we were intrigued to test our hypothesis to determine if the choroidal response after anti-VEGF therapy would differ between eyes with type 1 or 2 MNV and type 3 MNV.

To test our hypothesis, we used swept-source OCTA (SS-OCTA) imaging before and after anti-VEGF therapy in AMD eyes with MNV. The approach was similar to the one we used in the studies where we quantified choroidal parameters in eyes with PCV and proliferative diabetic retinopathy.^18^^,^^25^ In particular, we measured the choroidal thickness and the choroidal vascularity index (CVI) on full OCT scans with a 12 × 12-mm field of view rather than just relying one or a few B-scans in the subfoveal region.

In this report, we measured longitudinal choroidal changes at three time points: before exudation, at the onset of exudation, and after anti-VEGF therapy. In addition, we compared choroidal changes in eyes with type 1 or type 2 MNV with changes in eyes with type 3 MNV to determine if the site of origin for the MNV was associated with changes in the choroid.

## Methods

Patients were enrolled in an ongoing prospective SS-OCT imaging study (protocol number: 20120997) in accordance with the Declaration of Helsinki and the Health Insurance Portability and Accountability Act of 1996 and was approved by the Institutional Review Board of the University of Miami Miller School of Medicine. Informed consent was obtained from all patients. A post hoc retrospective review of this prospective observational SS-OCT imaging study included eyes from patients followed from April 2016 to December 2020. Subjects with a diagnosis of treatment-naïve eAMD and three different types of MNV were identified. Because this was a post hoc secondary analysis, no sample size calculations were performed since power analyses are designed for prospective studies and post-hoc power analyses in retrospective studies are inversely related to *P* values.^26^^,^^27^

Patients were followed as part of their routine clinical care at the Bascom Palmer Eye Institute and imaged using SS-OCTA scans (PLEX Elite 9000, Carl Zeiss Meditec, Dublin, CA). The instrument has a central wavelength of 1050 nm and a scanning rate of 100,000 A-scans per second. Theoretically, all eyes were imaged with the 12 × 12-mm scan pattern. This scan pattern consisted of 500 A-scans per B-scan at 500 B-scan positions (with each B-scan generated from two repetitions), resulting in a uniform spacing of 24 µm between adjacent A-scans in a 12 × 12-mm field of view. Of note, the actual scan area and spacing varies among different eyes owing to axial length.

The diagnosis of eAMD was based on the first visit with symptomatic exudation (day of exudation). The day of exudation was defined when symptomatic intraretinal fluid or subretinal fluid was first identified on OCT and treated with anti-VEGF therapy (aflibercept 2 mg [Regeneron Pharmaceuticals, Tarrytown, NY] or bevacizumab 1.25 mg [Genentech, South San Francsico, CA]). All eyes had SS-OCTA images acquired on the day of exudation and after two to four consecutive anti-VEGF injections. However, a subset of these eyes was followed as part of their routine clinical care before exudation developed and had SS-OCTA images acquired before the onset of exudation. In these eyes with SS-OCTA images before the exudation developed, the interval before the first treatment was matched with the interval chosen for the post-treatment interval. Exclusion criteria included low-quality SS-OCTA images with a signal strength of less than 7, motion artifacts, shadowing owing to media opacities, massive hemorrhages that obscured detection of the MNV lesion, high myopia (≥6.00 diopters), previous vitrectomy, and the presence of other concomitant retinal diseases.

The diagnosis of the different types of MNV was based on the SS-OCTA images on the day of exudation. Type 1 MNV was detected using a slab with segmentation boundaries along the RPE and Bruch's membrane, which identified neovascular lesions arising from the choriocapillaris and residing between the RPE and Bruch's membrane.^1^^,^^28^ Type 2 MNV was detected using a slab from the outer plexiform layer to the RPE, which identified neovascular lesions arising from the choriocapillaris that grow through the RPE into the subretinal space.^29^ Of note, in eyes with type 2 MNV, there was always type 1 MNV identified underneath the type 2 component. Because both type 1 and type 2 MNV arise from the choriocapillaris and connect with the choroid, these two types of MNV were grouped together for further comparison with type 3 MNV. In contrast, type 3 MNV, also known as retinal angiomatous proliferation, arises within the retina from the retinal circulation and grows toward the RPE. This type of MNV is often detected using a slab with segmentation boundaries between outer plexiform layer to RPE.^19^^,^^30^ To detect type 1, type 2, and type 3 MNV, we reviewed both the angiographic and structural images using both en face and B-scan images to confirm that both the flow and structural profiles were consistent with MNV. Two graders (M.S., J.L.) agreed on the classification and a senior grader (P.J.R,) adjudicated any disagreements.

The mean choroidal thickness and CVI measurements were obtained from the structural portion of the 12 × 12-mm SS-OCTA scans using an automatically validated algorithm as previously reported by Zhou et al.^31^^,^^32^ Optical attenuation correction was applied to the OCT scans to compensate the shadowing effect from retinal layers and to enhance the contrast of the choroidal layer, particularly at the choroidal–scleral interface.^33^ The Bruch's membrane and the choroidal–scleral interface were segmented automatically and manually corrected when necessary. The color-coded en face choroidal thickness maps were generated based on the distance between these two boundary segmentations. The mean choroidal thickness was calculated as the mean value of the choroidal thickness within a region. Choroidal vessels were segmented from the entire choroidal slab using Otsu's global thresholding method. En face CVI maps were generated by calculating the ratio between the number of pixels that belong to choroidal vessels and the number of pixels in the choroid for each A-scan position. To make sure the same area was compared among the visits, en face images from the visits of the same eye were registered using the retinal vessel features presented in OCTA retinal slabs. The optic nerve head was excluded in the mean choroidal thickness and CVI measurements. The mean choroidal thickness and CVI values were calculated in three regions within the 12 × 12-mm scans: a fovea-centered 5-mm circle, a fovea-centered 11-mm circle, and a 6 mm-wide rim derived from the outer boundary of the 5-mm circle to the outer boundary of the 11-mm circle. Owing to signal blockage resulting from exudation or pigment epithelial detachments on the underlying choroid, the areas where choroidal vessels could not be clearly detected at each visit were manually outlined to produce a binary mask, and this binary mask was used to exclude these areas from all visits for a given subject as previously described.^18^ Eyes were excluded from CVI analysis if the excluded area was more than 10% of the 5-mm circle's area.

Statistical analyses were performed using SAS version 9.4 (SAS Institute, Cary, NC) and R version 4.2.2.^34^ Data were summarized with mean and SD if applicable. Linear regression models with cluster-robust variance estimation^35^ to account for clustering of fellow eyes were used to assess statistical significance of differences in means. A two-sided *P* value of less than 0.05 was considered statistically significant.

## Results

A total of 41 eyes of 37 patients with treatment-naïve eAMD receiving anti-VEGF therapy were included. There were 24 eyes (59%) with type 1 MNV, 4 eyes (10%) with type 2 MNV, and 13 eyes (32%) with type 3 MNV. The mean age was 79.5 ± 7.5 years and 24 patients (65%) were women. All the 41 eyes had OCTA images taken on the day of exudation and at a post-treatment visit, and 27 eyes also had images from a pretreatment visit. The mean interval from the pretreatment visit to the day of exudation was 4.0 ± 1.8 months. The mean interval from the day of exudation to the post-treatment was 3.4 ± 0.8 months (*P* = 0.09). The mean number of anti-VEGF injections given was 3.0 ± 0.6. Thirty-two of the 41 eyes (78%) achieved a dry macula with resolution of intraretinal fluid and subretinal fluid on OCT after two to four anti-VEGF injections.

**Figure 1.**
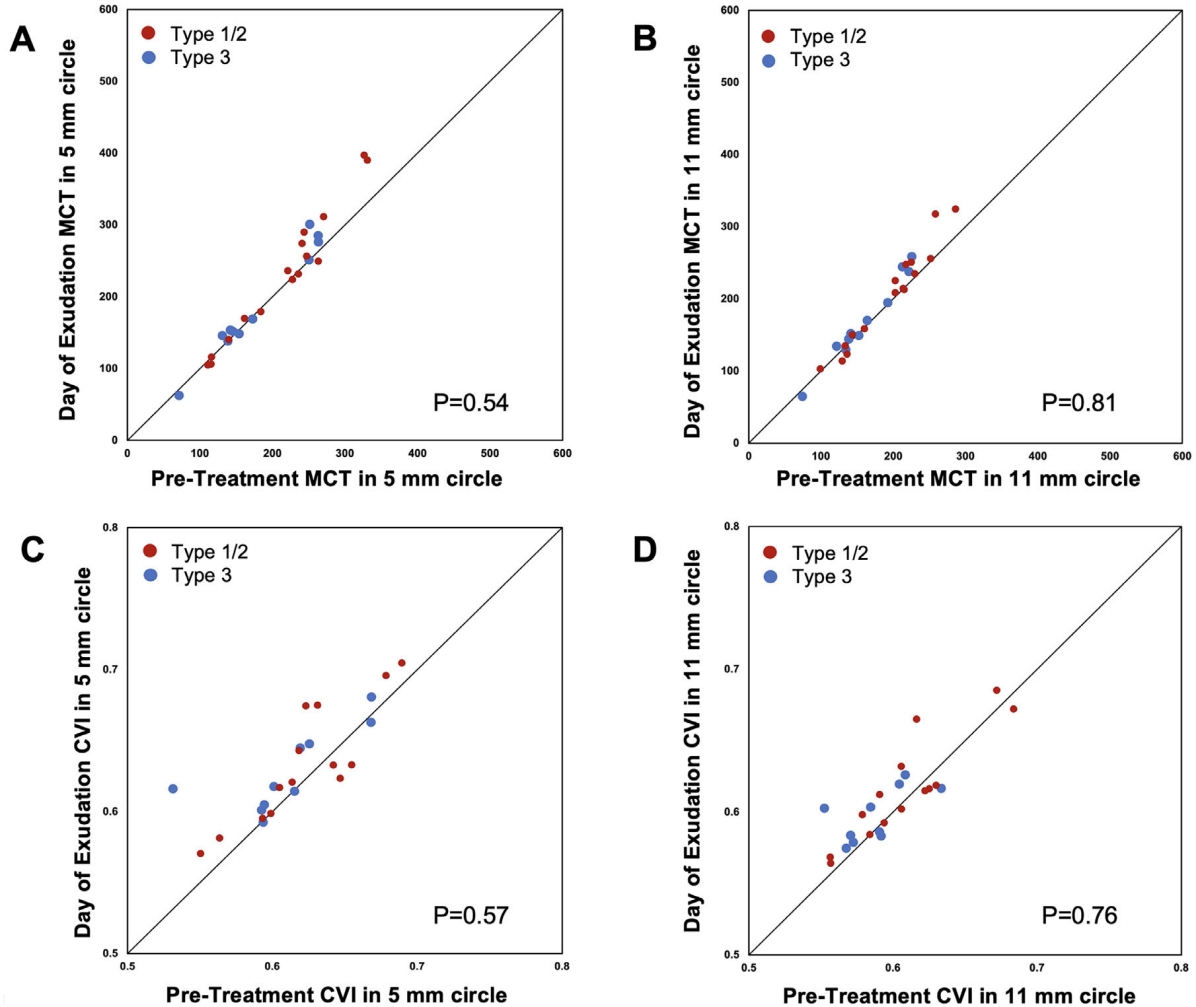
Scatter plots of mean choroidal thickness and CVI in 5-mm and 11-mm circles at the day of exudation vs. the pretreatment visit by different types of MNV. The mean choroidal thickness and CVI measurements increased on average from the pretreatment visit to the day of exudation. There were no significant differences in the changes of mean choroidal thickness or CVI measurements between type 1 or 2 MNV and type 3 MNV (all *P* ≥ 0.54). The diagonal line is a 1:1 reference line.

As shown in Table 1, the mean choroidal thickness measurements in the 5-mm and 11-mm circles increased by 12.7 ± 22.8 µm (*P* = 0.03) and 10.1 ± 17.2 µm (*P* = 0.02), respectively, from the pretreatment visit to the day of exudation for all eyes without considering MNV types. The CVI measurements in the 5-mm and 11-mm circles increased by 0.014 ± 0.023 (*P* = 0.009) and 0.009 ± 0.017 (*P* = 0.02), respectively, during the same time interval. From the pretreatment visit to the day of exudation, there were no significant differences in the increase in mean choroidal thickness and CVI measurements between eyes with type 1 or 2 MNV and type 3 MNV (all *P* ≥ 0.54) (Fig. 1, Table 2).

**Table 1.** Pretreatment to Day of Exudation Mean Choroidal Thickness and CVI in All Eyes, With no Consideration for MNV Type

	5-mm Circle	11-mm Rim^*^	11-mm Circle
Pretreatment mean choroidal thickness, µm (*N* = 27)	200.7 ± 69.5	175.6 ± 49.4	180.8 ± 52.9
Day of exudation mean choroidal thickness, µm (*N* = 27)	213.4 ± 86.4	184.8 ± 60.7	191 ± 65.6
Increase in mean choroidal thickness, µm	12.7 ± 22.8	9.2 ± 16.2	10.1 ± 17.2
*P* value	0.03	0.02	0.02
Pretreatment CVI (*N* = 24)	0.617 ± 0.039	0.594 ± 0.033	0.600 ± 0.033
Day of exudation CVI (*N* = 24)	0.631 ± 0.036	0.601 ± 0.031	0.608 ± 0.032
Increase in CVI	0.014 ± 0.023	0.007 ± 0.017	0.009 ± 0.017
*P* value	0.009	0.05	0.02

Values are mean ± SD.

* An 11-mm rim is a 6-mm-wide rim between the outer boundary of the 5-mm circle and the outer boundary of the 11-mm circle.

**Table 2.** Increase in Mean Choroidal Thickness and CVI From Pretreatment to Day of Exudation by MNV Type

MNV Type	5-mm Circle	11-mm Rim^*^	11-mm Circle
Increase in mean choroidal thickness, µm			
Type 1 or 2 (*N* = 16)	15.3 ± 26.4	10 ± 18.3	10.9 ± 19.6
Type 3 (*N* = 11)	8.8 ± 16.5	8.1 ± 13.3	8.9 ± 13.7
*P* value	0.54	0.8	0.81
Increase in CVI			
Type 1 or 2 (*N* = 14)	0.011 ± 0.021	0.006 ± 0.018	0.008 ± 0.017
Type 3 (*N* = 10)	0.017 ± 0.026	0.008 ± 0.017	0.01 ± 0.019
*P* value	0.57	0.84	0.76

Values are mean ± SD.

* An 11-mm rim is a 6-mm-wide rim between the outer boundary of the 5-mm circle and the outer boundary of the 11-mm circle.

**Figure 2.**
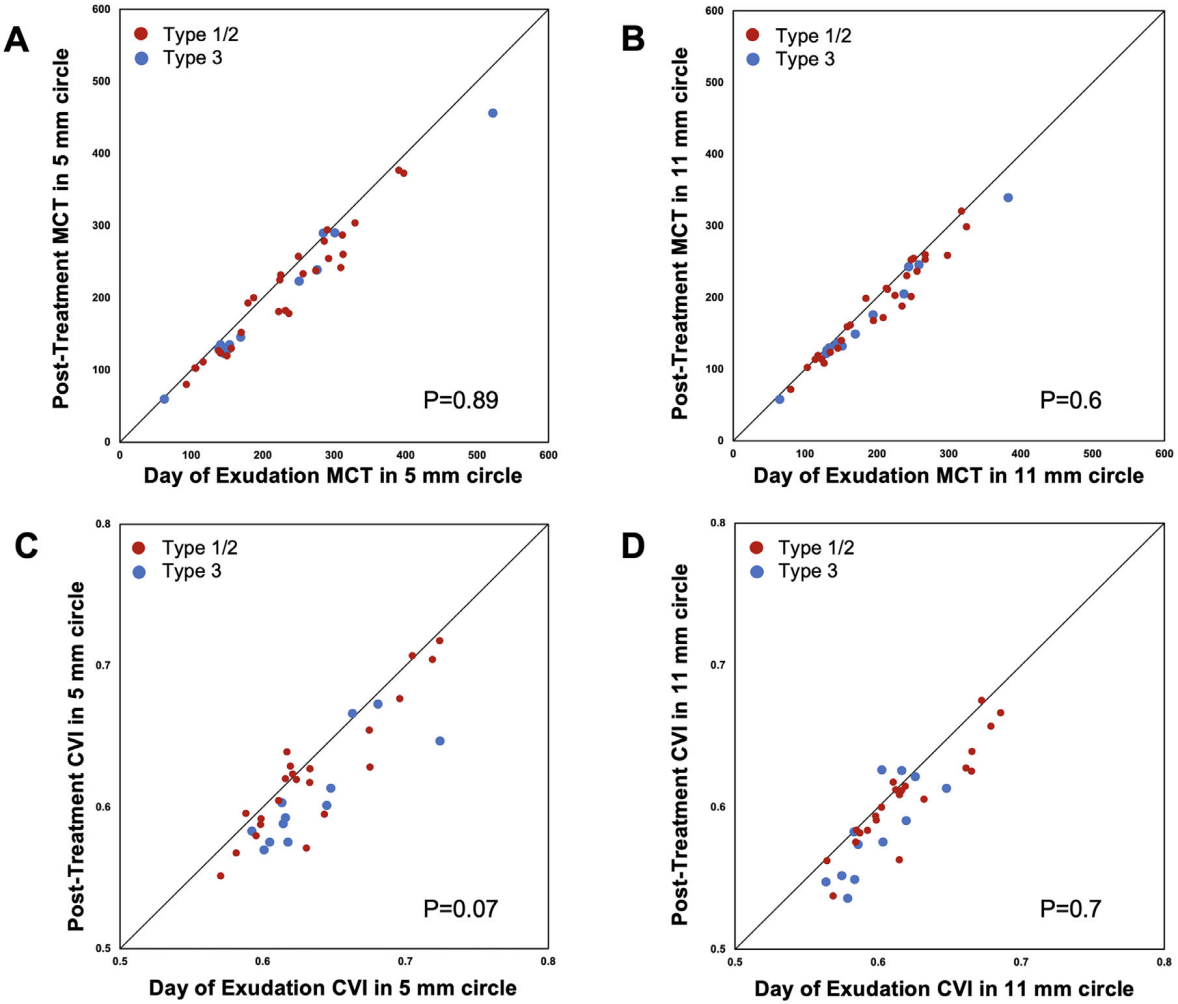
Scatter plots of mean choroidal thickness and CVI in 5-mm and 11-mm circles at the post-treatment visit versus the day of exudation by different types of MNV. The mean choroidal thickness and CVI measurements decreased on average from the day of exudation to the post-treatment visit. There were no significant differences in the changes of mean choroidal thickness or CVI measurements between type 1 or 2 MNV and type 3 MNV (all *P* ≥ 0.07). The diagonal line is a 1:1 reference line.

On the day of exudation, the mean choroidal thickness measurements in the 5-mm circle of eyes with type 1 or 2 MNV and type 3 MNV were 227.8 ± 85.2 µm and 211.2 ± 117.3 µm, respectively (*P* = 0.68). The CVI measurements in the 5-mm circle in eyes with type 1 or 2 MNV and type 3 MNV were 0.64 ± 0.04 and 0.63 ± 0.04, respectively (*P* = 0.99). As shown in Table 3, the mean choroidal thickness measurements in the 5-mm and 11-mm circles decreased by 19.5 ± 20.3 µm (*P* < 0.001) and 13.1 ± 14.7 µm (*P* < 0.001), respectively, from the day of exudation to the post-treatment visit for all eyes. The CVI measurements in the 5-mm and 11-mm circles decreased by 0.017 ± 0.021 (*P* < 0.001) and 0.014 ± 0.017 (*P* < 0.001). There were no overall statistically significant differences in the decrease in mean choroidal thickness and CVI measurements from the day of exudation to the post-treatment visit when comparing the type 1 or 2 and type 3 MNV eyes (all *P* ≥ 0.07) (Fig. 2, Table 4).

**Table 3.** Day of Exudation to Post-Treatment Mean Choroidal Thickness and CVI in All Eyes, With no Consideration for MNV Type

	5-mm Circle	11-mm Rim^*^	11-mm Circle
Day of exudation mean choroidal thickness, µm (*N* = 41)	222.6 ± 95.3	188.2 ± 66.8	195.2 ± 71.6
Post-treatment mean choroidal thickness, µm (*N* = 41)	203.1 ± 88.5	176.5 ± 62.5	182.1 ± 67.2
Decrease in mean choroidal thickness, µm	19.5 ± 20.3	11.7 ± 14.3	13.1 ± 14.7
*P* value	<0.001	<0.001	<0.001
Day of exudation CVI (*N* = 34)	0.635 ± 0.042	0.605 ± 0.034	0.612 ± 0.034
Post-treatment CVI (*N* = 34)	0.618 ± 0.043	0.592 ± 0.035	0.598 ± 0.035
Decrease in CVI	0.017 ± 0.021	0.013 ± 0.019	0.014 ± 0.017
*P* value	<0.001	<0.001	<0.001

Values are mean ± SD.

* An 11-mm rim is a 6-mm-wide rim between the outer boundary of the 5-mm circle and the outer boundary of the 11-mm circle.

**Table 4.** Decrease in Mean Choroidal Thickness and CVI From Day of Exudation to Post-treatment by MNV Type

MNV Type	5-mm Circle	11-mm Rim^*^	11-mm Circle
Decrease in mean choroidal thickness, µm			
Type 1 or 2 (*N* = 28)	19.2 ± 21.6	10.8 ± 15.3	12.3 ± 15.8
Type 3 (*N* = 13)	20.1 ± 17.8	13.7 ± 12.0	14.7 ± 12.2
*P* value	0.89	0.5	0.6
Decrease in CVI			
Type 1 or 2 (*N* = 22)	0.012 ± 0.019	0.014 ± 0.017	0.013 ± 0.015
Type 3 (*N* = 12)	0.028 ± 0.021	0.012 ± 0.022	0.016 ± 0.02
*P* value	0.07	0.83	0.7

Values are mean ± SD.

* An 11-mm rim is a 6-mm-wide rim between the outer boundary of the 5-mm circle and the outer boundary of the 11-mm circle.

**Figure 3.**
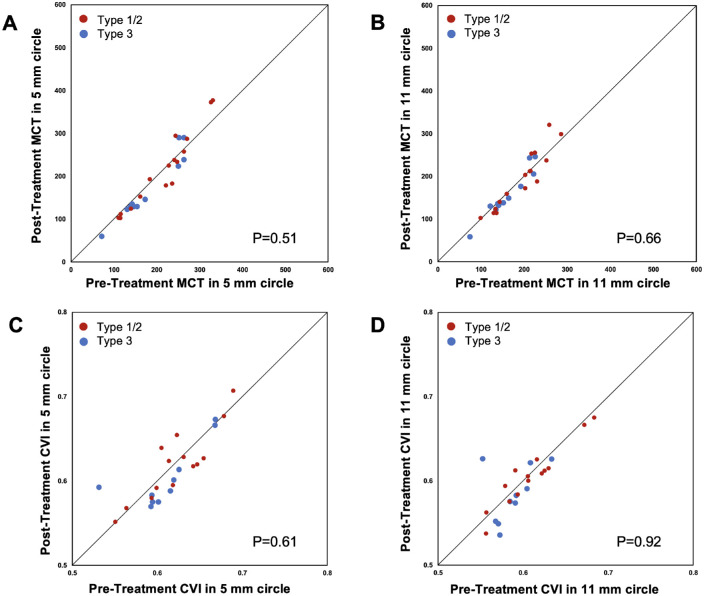
Scatter plots of mean choroidal thickness and CVI in 5-mm and 11-mm circles at the post-treatment visit versus the pretreatment visit by different types of MNV. The mean choroidal thickness and CVI measurements did not change significantly from pretreatment to post-treatment. There were no significant differences in the changes of mean choroidal thickness or CVI measurements between type 1 or 2 MNV and type 3 MNV (all *P* ≥ 0.51). The diagonal line is a 1:1 reference line.

Among 27 eyes with a visit before exudation developed, the pretreatment and post-treatment choroidal measurements were compared. As shown in Table 5, the mean choroidal thickness and CVI measurements after anti-VEGF therapy were not significantly different from those before exudation developed (all *P* ≥ 0.38). The data in Figure 3 and Table 6 show that between eyes with type 1 or 2 MNV and type 3 MNV, there were no significant differences in the changes of mean choroidal thickness or CVI measurements from the pretreatment to the post-treatment visit (all *P* ≥ 0.51).

**Table 5.** Pretreatment to Post-Treatment Mean Choroidal Thickness and CVI in All Eyes, With no Consideration for MNV Type

	5-mm Circle	11-mm Rim^*^	11-mm Circle
Pretreatment mean choroidal thickness, µm (*N* = 27)	200.7 ± 69.5	175.6 ± 49.4	180.8 ± 52.9
Post-treatment mean choroidal thickness, µm (*N* = 27)	197.2 ± 84.5	174.0 ± 59.1	179.2 ± 64.3
Change in mean choroidal thickness, µm	–3.5 ± 26.2	–1.6 ± 21.6	–1.6 ± 22.1
*P* value	0.57	0.71	0.74
Pretreatment CVI (*N* = 24)	0.617 ± 0.039	0.594 ± 0.033	0.600 ± 0.033
Post-treatment CVI (*N* = 24)	0.613 ± 0.040	0.591 ± 0.036	0.596 ± 0.036
Change in CVI	–0.004 ± 0.023	–0.003 ± 0.021	–0.003 ± 0.020
*P* value	0.38	0.45	0.38

Values are mean ± SD.

* An 11-mm rim is a 6-mm-wide rim between the outer boundary of the 5-mm circle and the outer boundary of the 11-mm circle.

**Table 6.** Change in Mean Choroidal Thickness and CVI From Pretreatment to Post-Treatment by MNV Type

MNV Type	5-mm Circle	11-mm Rim^*^	11-mm Circle
Change in mean choroidal thickness, µm			
Type 1 or 2 (*N* = 16)	–0.1 ± 29.0	0.2 ± 25.5	0.1 ± 25.7
Type 3 (*N* = 11)	–8.4 ± 21.8	–4.3 ± 15.1	–4.2 ± 16.4
*P* value	0.51	0.62	0.66
Change in CVI			
Type 1 or 2 (*N* = 14)	–0.002 ± 0.019	–0.003 ± 0.014	–0.003 ± 0.011
Type 3 (*N* = 10)	–0.007 ± 0.025	–0.003 ± 0.030	–0.004 ± 0.029
*P* value	0.61	0.96	0.92

Values are mean ± SD.

* An 11-mm rim is a 6-mm-wide rim between the outer boundary of the 5-mm circle and the outer boundary of the 11-mm circle.

**Figure 4.**
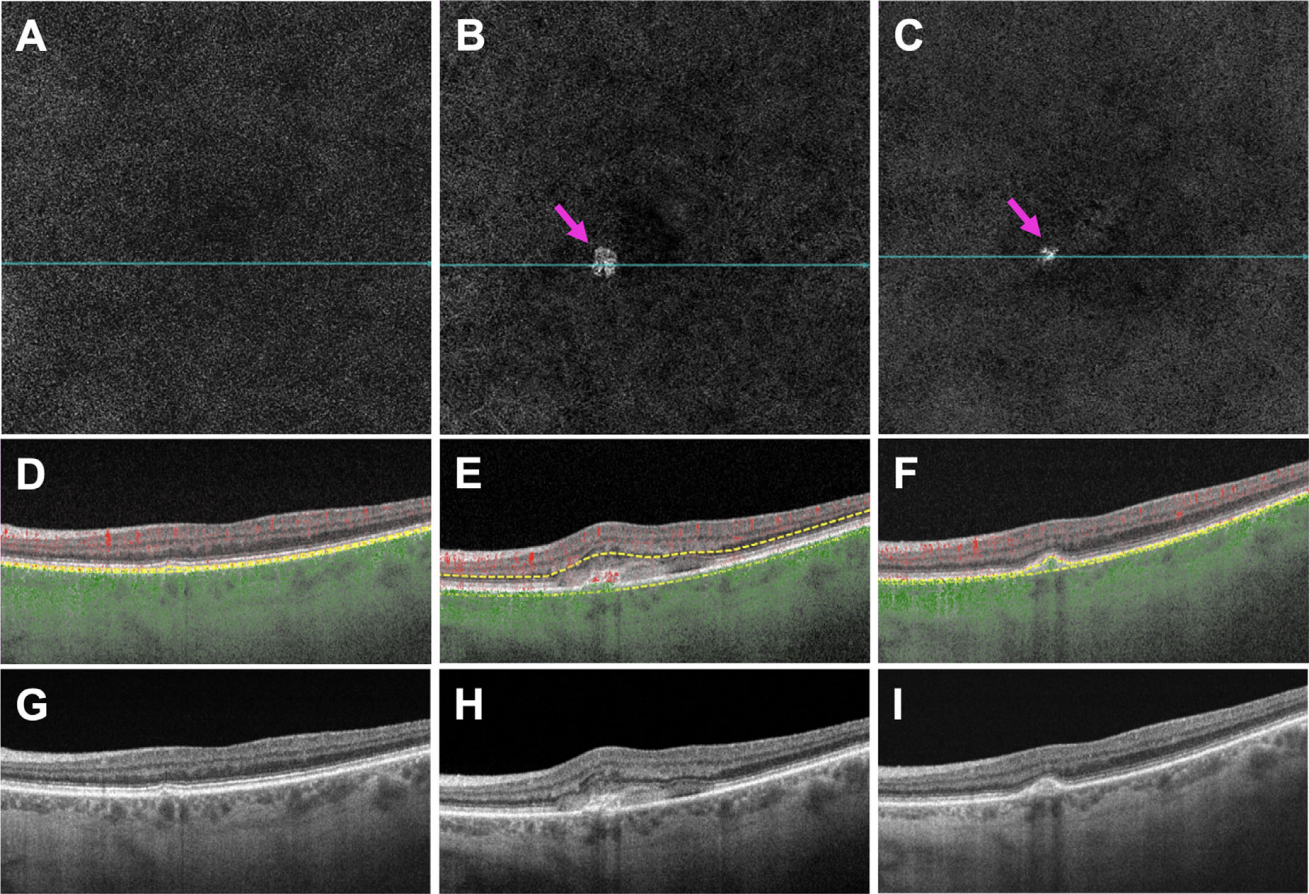
Images of a treatment-naïve eye with AMD that developed type 1 and type 2 MNV: at the pretreatment visit (**A,** **D,** **G**) without MNV or any exudation; at the day of exudation visit (**B,** **E,** **H**), showing the new type 1 and type 2 MNV (*pink arrow*) with subretinal hyper-reflective material (SHRM). (**E,** **H**) Three months after the day of exudation visit and after three monthly anti-VEGF injections (**C,** **F,** **I**), showing regression of the MNV lesion (*pink arrow*) and no exudation. (**A–C**) En face flow images. (**D–F**) B-scans with flow and segmentation lines (*yellow*) corresponding with the blue lines in (**A–C**). (**G–I**) The same structural B-scans as in (**D–F**) without flow or segmentation lines.

**Figure 5.**
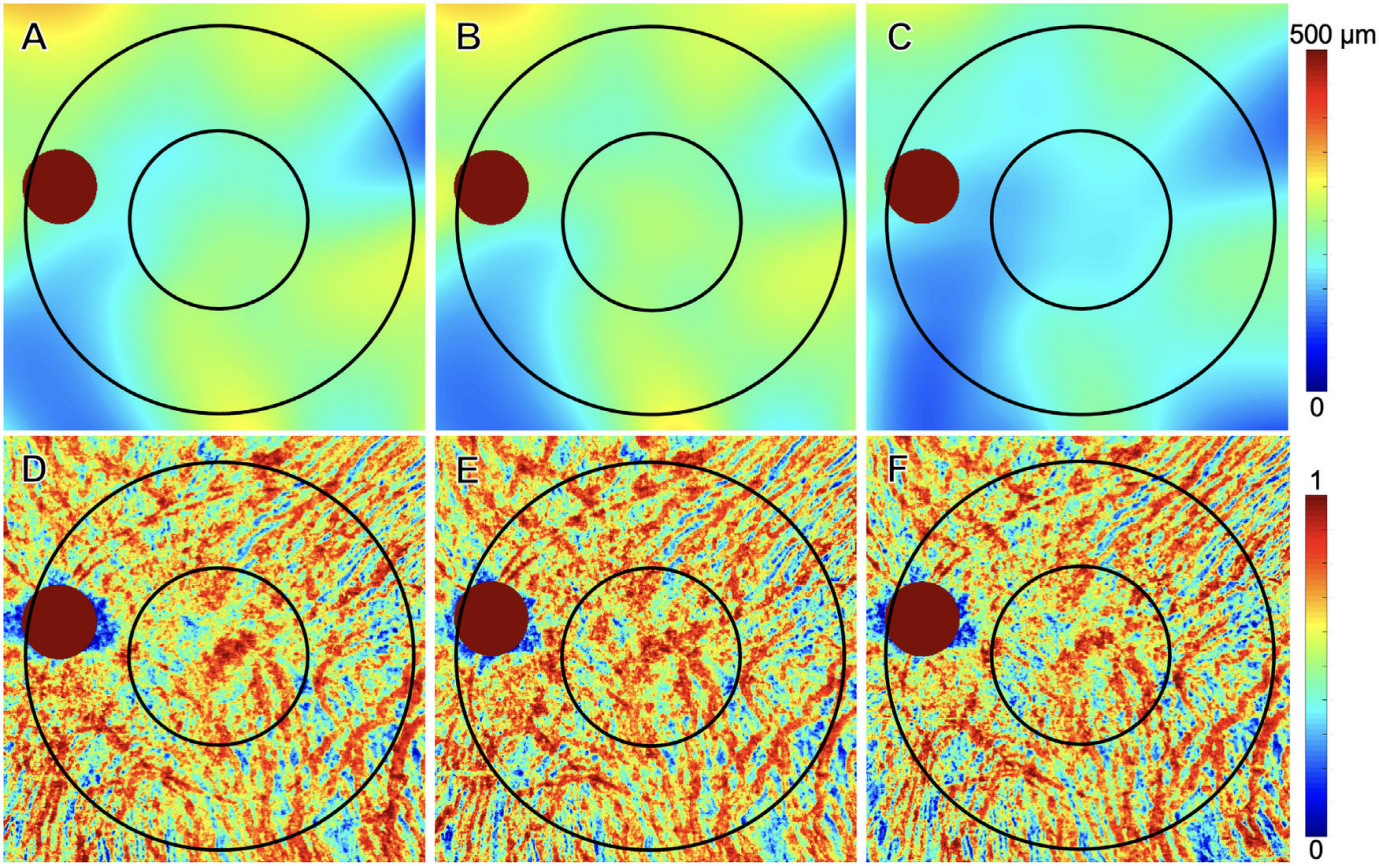
Choroidal changes after anti-VEGF therapy of a treatment-naïve eye with AMD that developed type 1 and type 2 MNV. The same eye as in Figure 4 showed an increase in mean choroidal thickness and CVI measurements when exudation developed, followed by a decrease in these choroidal measurements after anti-VEGF therapy. The 5-mm and 11-mm circles centered on the fovea are shown. Optic nerve head regions are indicated using the red circles and were excluded in measurements. (**A–C**) The mean choroidal thickness maps at pretreatment visit, day of exudation visit and post-treatment visit. (**D–F**) CVI maps at pretreatment visit, day of exudation, visit and post-treatment visit.

**Figure 6.**
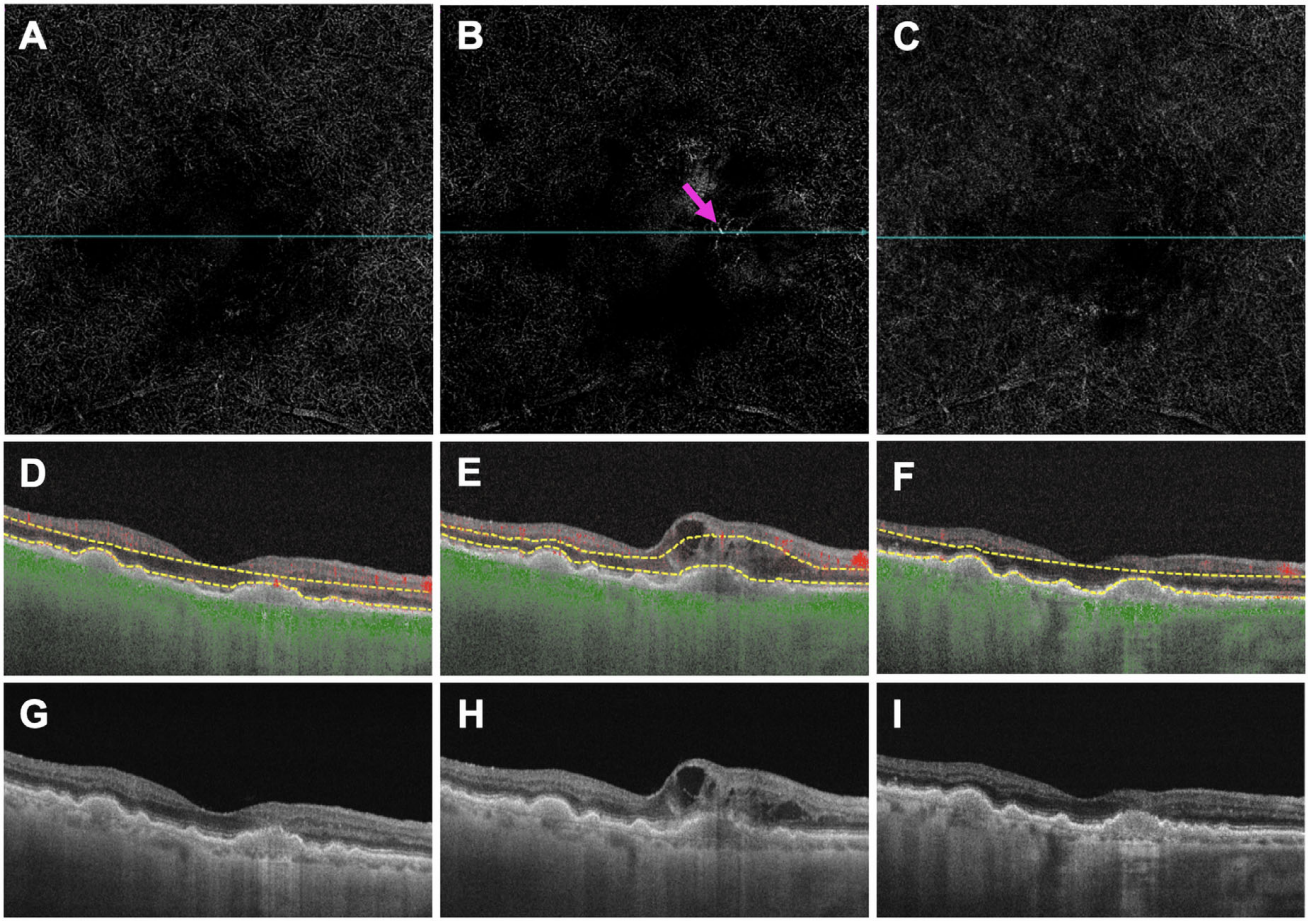
Images of a treatment-naïve eye with AMD that developed type 3 MNV: at the pretreatment visit (**A,** **D,** **G**), showing a preclinical type 3 MNV but no exudation; at the day of exudation visit (**B,** **E,** **H**), showing a new type 3 MNV (*pink arrow*) with intraretinal fluid; 2 months after the day of exudation visit and after two monthly anti-VEGF injections (**C,** **F,** **I**), showing complete resolution of the type 3 MNV lesion and no exudation. (**A–C**) En face flow images. (**D–F**) B-scans with flow and segmentation lines (*yellow*) corresponding to the blue lines in (**A–C**). (**G–I**) The same structural B-scans as in (**D–F**) without flow or segmentation lines.

**Figure 7.**
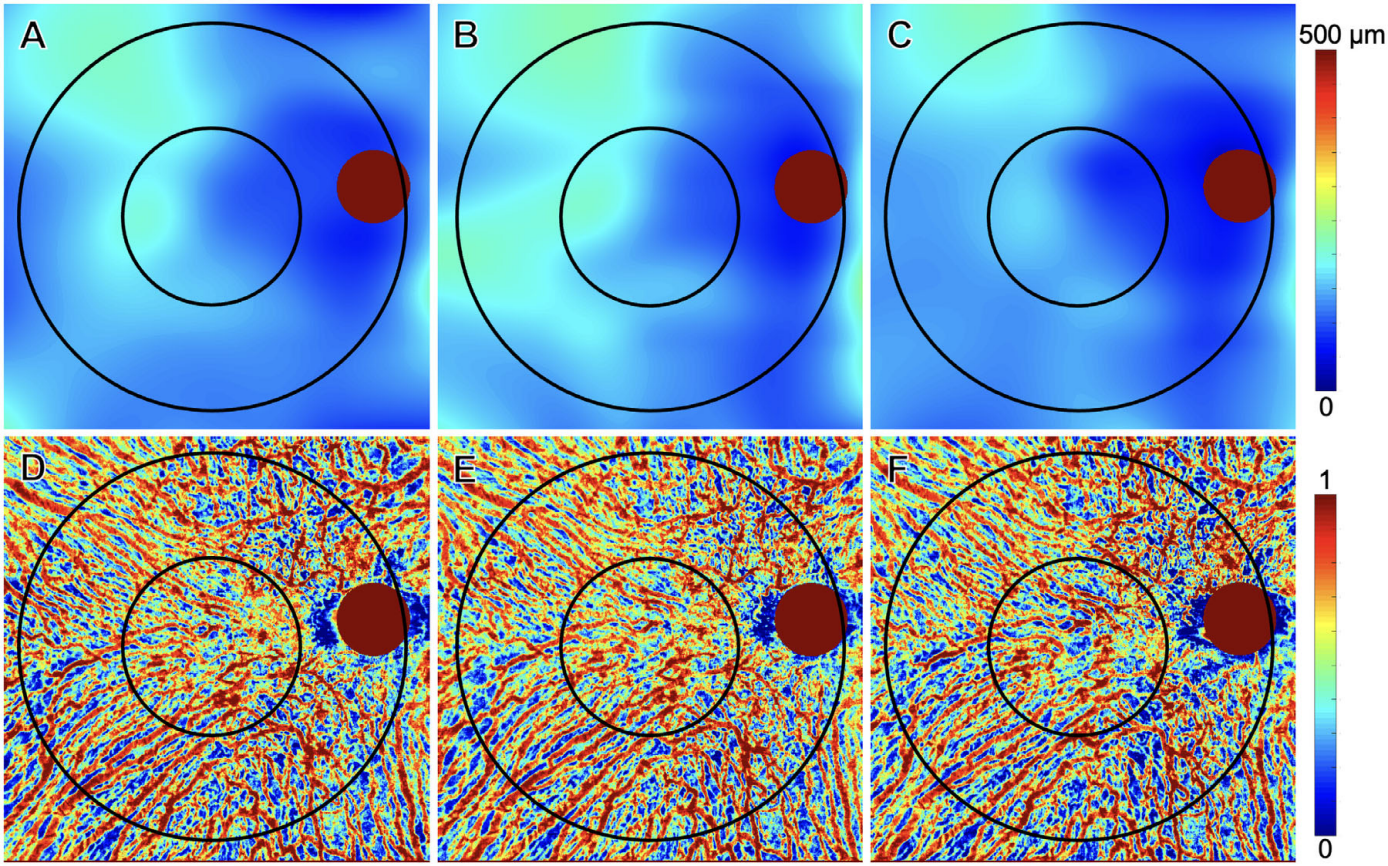
Choroidal changes after anti-VEGF therapy of a treatment-naïve eye with AMD that developed type 3 MNV. The same eye as in Figure 6 showed an increase in mean choroidal thickness and CVI measurements when exudation developed, followed by a decrease in these choroidal measurements after anti-VEGF therapy. The 5-mm and 11-mm circles centered on the fovea are shown. Optic nerve head regions are indicated using the red circles and were excluded in measurements. (**A–C**) Mean choroidal thickness maps at pretreatment visit, day of exudation visit and post-treatment visit. (**D–F**) CVI maps at pretreatment visit, day of exudation visit, and post-treatment visit.

Figures 4–7 show two representative eyes which developed a type 1 or 2 MNV lesion (Figs. 4 and 5) and a type 3 MNV lesion (Figs. 6 and 7) along with their treatment response after anti-VEGF therapy. Both eyes showed an increase in choroidal thickness and vascularity once exudation developed, followed by a decrease in choroidal thickness and vascularity after anti-VEGF therapy. Overall, mean choroidal thickness and CVI measurements after treatment reverted to their baseline values once the macula became fluid free after receiving anti-VEGF therapy.

## Discussion

In this study, we found that mean choroidal thickness and CVI measurements increased from pretreatment to the day of exudation, and once anti-VEGF therapy was initiated, the measurements decreased from the day of exudation to the post-treatment visit. Overall, in those eyes with pretreatment and post-treatment visits, mean choroidal thickness and CVI measurements after treatment reverted to the values before exudation developed. These findings before and at the time of exudation are consistent with the previous findings by Park et al.^16^ They showed that choroidal thickness increased significantly at the visit when the exudation was first detected compared with the visit 6 months prior when there was no exudation. Moreover, they showed that the choroid was thicker at 3 months before the exudation developed compared with 6 months before the exudation, suggesting that the choroid continues to thicken before the onset of exudation. When comparing changes after anti-VEGF therapy, other studies have shown that choroidal thickness and vascularity significantly decreased after anti-VEGF treatment over a 3-month period and lasted for 12 months.^6^^,^^11^^,^^36^ However, we addressed the longitudinal changes in the choroid from the time of no exudation, to the onset of exudation, and then after anti-VEGF therapy. We found that post-treatment choroidal measurements reverted to the pretreatment level when there was no exudation. This finding may address some of the concern surrounding the thinning of the choroid associated with anti-VEGF therapy. Because the choroid is already thickened when the exudation develops and when the treatment is initiated, the conclusion that the choroid becomes thinner after anti-VEGF therapy when compared with the day of exudation seems to be irrelevant. Instead, based on our results, the decrease in the choroidal thickness after anti-VEGF therapy reverses the increase in thickness that developed before the onset of exudation. Furthermore, histopathological studies have shown that the loss of choroidal vasculature and choroidal thinning in neovascular AMD develops even before the onset of exudation.^13^^,^^37^ Thus, the thinning of the choroid in the long term is likely a part of the degenerative progression of the disease or a feature of decreased choroidal perfusion and not due to the short-term response of the choroid to anti-VEGF therapy, which only returns the choroid to its appearance before exudation developed.^38^^–^^40^

In our previous study of eyes with PCV undergoing anti-VEGF therapy, we found that the mean choroidal thickness decreased, but the CVI increased, after treatment.^18^ The discrepancy in the change of CVI between our two studies may indicate that the choroid plays a different role in the pathogenesis of PCV and AMD or that the flow properties within the PCV lesions are greater compared with MNV in AMD or that the change in CVI is more subtle in these eAMD eyes, suggesting that additional cases are needed. Because the mean choroidal thickness is mainly determined by the choroidal vessel volume,^32^ and the mean choroidal thickness decreased in both AMD and PCV, it would seem that the mean choroidal vessel volume decreases in both AMD and PCV after anti-VEGF therapy. The CVI is the ratio of the choroidal vessel volume to the total choroidal volume, which consists of both the choroidal vessel and the choroidal stroma. An increase in CVI found in PCV after treatment would suggest a greater decrease in choroidal stroma than vessel volume, which may result from greater resorption of the excess stromal transudation than in AMD eyes with MNV. In comparison, the decrease in CVI after treatment in AMD suggests that there may be less stromal transudation in the choroid. Our explanation is supported by studies using indocyanine green angiography showing that choroidal vascular hyperpermeability occurs more frequently in eyes with PCV than typical eAMD, and choroidal vascular remodeling may be driven by different stimuli in PCV versus typical eAMD.^41^^,^^42^ Another possibility is that the proangiogenic milieu is much greater in eyes with PCV, and anti-VEGF therapy has a more dramatic choroidal impact. In addition, PCV tends to occur in a younger population than typical MNV in AMD, and the choroid may be thicker and more dynamic in a younger eye than an older eye.

Of note, our results did not show a significant difference in the change in the mean choroidal thickness or CVI in eyes with type 1 or 2 MNV versus type 3 MNV, despite their different connectivity with the choroid. These results suggest that the changes of the choroid in eAMD are mainly driven by the overall angiogenic milieu of the eye, with VEGF being the likely causative agent, given the choroid's response to anti-VEGF therapy. We had expected to find a difference in choroidal changes between type 1 or 2 and type 3 MNV after anti-VEGF therapy, given the connectivity of type 1 or 2 MNV to the choroid and the possibility that these lesions could serve as arteriovenous shunts between the choroidal arterial and venous circulation. If this were the case, then anti-VEGF therapy would decrease the flow within the type 1 or 2 MNV shunt and more markedly impact the choroidal thickness and vascularity compared with the type 3 MNV, but this result was not observed. One possibility is that a difference might exist, but the proangiogenic milieu or the flow is less in type 1 or 2 MNV compared with PCV lesions and more patients are needed to detect this smaller difference. Another possibility is that small changes in older eyes are more difficult to detect given that the choroidal thickness is less at baseline and AMD may have impacted the choroidal architecture so that it is less affected by changes in perfusion pressures.

The strength of this study is that we measured the choroid parameters using 12 × 12 volumetric OCTA scans, which consider the entirety of the macular region, compared with most the current studies, which have been limited to subfoveal measurement of the choroid. Additionally, we included three types of MNV and compared the changes of the choroid in type1 or 2 MNV and type 3 MNV based on their different origin from the choroid or from the retina. Hence, our study can provide useful information for clinicians and investigators to understand the choroidopathy associated with different types of MNV, especially type 3 MNV. Most important, we followed the choroidal changes longitudinally at three time points, from before developing exudation to the exudation visit and after treatment, filling the current knowledge gap of how the choroid changes after treatment compared with an earlier time point before any exudation.

The limitations of our study include the small sample size, especially of type 2 and type 3 MNV, which can result in large SDs. There is a distinct possibility that differences in the changes in the mean choroidal thickness and CVI in eyes with type 1 or 2 MNV and type 3 MNN may exist, but we were unable to achieve statistical significance. Second, not all the treatment-naïve eyes included in our study had a pretreatment visit before the onset of exudation, because these patients were referred to the retina specialist when symptoms developed and not before. Of note, variability in the lateral scale of the images, particularly in the absence of axial length corrections, can introduce errors in the choroidal measurements.^43^ However, this limitation should not significantly impact our conclusions, because we compared the longitudinal choroidal changes in the same eye and none of the eyes had any of the characteristic features associated with high myopia or a history of refractive error of more than 6 diopters. In addition, the diurnal variations may have affected some of the changes in choroidal measurements^44^; however, it seems unlikely that our results are due to these variations and may have been even better if all the scans were performed at the same time at each visit.^45^ Last, although there might be a difference in the choroidal response based on the type of anti-VEGF agents used, we decided to focus on the class of drugs and elected not to divide the patient population into smaller subgroups for the purpose of comparing the drugs.

In summary, mean choroidal thickness and CVI measurements in treatment-naïve eAMD eyes increased from pretreatment to the day of exudation and then decreased from the day of exudation to the post-treatment visit. The post-treatment mean choroidal thickness and CVI measurements were not significantly different from the pretreatment visit before exudation. Moreover, eyes with type 1 or 2 MNV and type 3 MNV had similar changes in the mean choroidal thickness and CVI measurements. These findings suggest that the choroid increases in thickness in response to the overexpression of proangiogenic factors, most likely VEGF, and reverts to the pre-exudation state after anti-VEGF therapy regardless of the MNV type.
